# Autocatalytic sets in *E. coli* metabolism

**DOI:** 10.1186/s13322-015-0009-7

**Published:** 2015-04-01

**Authors:** Filipa L Sousa, Wim Hordijk, Mike Steel, William F Martin

**Affiliations:** Institute of Molecular Evolution, Heinrich Heine Universität, Düsseldorf, Germany; SmartAnalytiX.com, Lausanne, Switzerland; Allan Wilson Centre Molecular Ecology and Evolution, University of Canterbury, Christchurch, New Zealand

**Keywords:** Autocatalytic networks, Origin of life, Metabolic network

## Abstract

**Background:**

A central unsolved problem in early evolution concerns self-organization towards higher complexity in chemical reaction networks. In theory, autocatalytic sets have useful properties to help model such transitions. Autocatalytic sets are chemical reaction systems in which molecules belonging to the set catalyze the synthesis of other members of the set. Given an external supply of starting molecules – the food set – and the conditions that (i) all reactions are catalyzed by at least one molecule, and (ii) each molecule can be constructed from the food set by a sequence of reactions, the system becomes a reflexively autocatalytic food-generated network (RAF set). Autocatalytic networks and RAFs have been studied extensively as mathematical models for understanding the properties and parameters that influence self-organizational tendencies. However, despite their appeal, the relevance of RAFs for real biochemical networks that exist in nature has, so far, remained virtually unexplored.

**Results:**

Here we investigate the best-studied metabolic network, that of *Escherichia coli*, for the existence of RAFs. We find that the largest RAF encompasses almost the entire *E. coli* cytosolic reaction network. We systematically study its structure by considering the impact of removing catalysts or reactions. We show that, without biological knowledge, finding the minimum food set that maintains a given RAF is NP-complete. We apply a randomized algorithm to find (approximately) smallest subsets of the food set that suffice to sustain the original RAF.

**Conclusions:**

The existence of RAF sets within a microbial metabolic network indicates that RAFs capture properties germane to biological organization at the level of single cells. Moreover, the interdependency between the different metabolic modules, especially concerning cofactor biosynthesis, points to the important role of spontaneous (non-enzymatic) reactions in the context of early evolution.

**Graphical Abstract:**
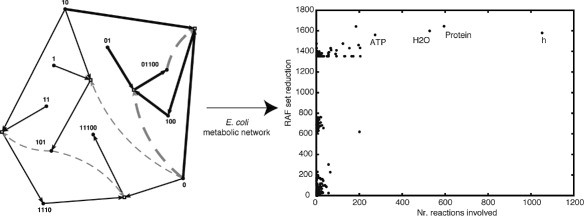
*E. coli* metabolic network in the context of autocatalytic sets.

**Electronic supplementary material:**

The online version of this article (doi:10.1186/s13322-015-0009-7) contains supplementary material, which is available to authorized users.

## Background

Autocatalytic sets were initially proposed as chemical reaction networks with intrinsic properties that could promote a natural rudimentary selection process en route towards self organization in chemical evolution [1-4]. Until now, autocatalytic sets were mostly theoretical constructs, with only a few artificially designed and constructed examples in real chemistry [5-10]. However, with one exception [11], actual (evolved) biological networks have not been studied explicitly in the context of autocatalytic sets. Here, we take a step in this direction. In particular, we apply a formal framework for autocatalytic sets, known as RAF theory (see Experimental below), to the best-studied metabolic network, that of *E. coli*.

In order to perform such an analysis, we had to modify the available *E. coli* metabolic network data [12] to conform to the formal RAF framework. For example, since most of the metabolic reactions dealing with carbon and energy metabolism occur within the cytoplasm, the periplasmic reactions as well as transport reactions between the environment, periplasm and cytoplasm were discarded. The exceptions are a few reactions annotated as oxidative phosphorylation, for example cytochrome *c* reduction and oxidation, which were kept. Also, to apply the RAF algorithm to *E. coli* metabolism, the network has to be expressed in terms of molecules-reactions-catalysts and, when possible, having the catalyst of each reaction expressed in terms of the cofactors present in the enzyme that catalyzes the reaction. In our definition, we include as cofactors all non-protein chemical compounds that are present and/or assist a biochemical transformation. Thus, metal ions, iron-sulfur centers, as well as organic molecules such as flavins or quinones were considered as cofactors. Moreover, if a cofactor like the ones above mentioned is a reactant within a chemical reaction, it would be considered a catalyst of that reaction as well. We did not make any distinction between quinone types, flavin-species or NAD-species partially because of lack of information within annotations and also due to possible promiscuity between the use of analog cofactors. This approach is in agreement with the view that cofactors themselves, metals or even simple amino acids were the initial catalysts of biological reactions [13-19]. This principle can be illustrated with the example of the cofactor pyridoxal phosphate (PLP): in a comparison of 2-aminoisobutyrate decarboxylation reactions catalyzed by a PLP-dependent enzyme, the enzyme-PLP complex was shown to increase the reaction rate 10 ^18^-fold, whereas in the absence of the enzyme, PLP alone increased the rate of decarboxylation by 10 ^10^-fold [20]. As for invoking the role of metals as early catalysts, the continuous abiotic production of methane and formate by serpentinization reactions shows a common trail that connects biology with geochemical occurring reactions whose metal-based “catalysts” embedded in minerals resemble the metal centers found in modern enzymes [21]. This support the view shared by many of the important role of metals in early evolution [14,16,22,23].

Having thus prepared the *E. coli* metabolic network for analysis, we applied the RAF framework to search for autocatalytic sets within it, studied their structure, and performed sensitivity analyses in terms of importance of individual molecules, reactions, and catalysts.

## Experimental

### Autocatalytic sets

The concept of autocatalytic sets was introduced several decades ago [2-4], and formalized more recently with RAF theory [24-26]. It aims to model life as a functionally closed, self-sustaining reaction system. We briefly review the main definitions and results of RAF theory here.

First, a *chemical reaction system* (CRS) is defined as a tuple $Q=\{X,{\mathcal {R}},C\}$ consisting of a set of chemical species (molecule types) *X*, a set of chemical reactions , and a catalysis set *C* indicating which molecule types catalyze which reactions. Next, the notion of a food set *F*⊂*X* is included, which is a subset of molecule types that are assumed to be freely available from the environment. Finally, an *autocatalytic set* (or RAF set) is defined as a subset ${\mathcal {R}}' \subseteq {\mathcal {R}}$ of reactions (and associated molecule types) which is:

1. *Reflexively Autocatalytic* (RA): each reaction $r \in {\mathcal {R}}'$ is catalyzed by at least one molecule type involved in ${\mathcal {R}}'$, and
2. *Food-generated* (F): all reactants in ${\mathcal {R}}'$ can be created from the food set *F* by using a series of reactions only from ${\mathcal {R}}'$ itself.

This definition captures the notion of a functionally closed (RA) and self-sustaining (F) reaction network. A formal mathematical definition of RAF sets is provided in [25,27], including an algorithm for finding RAF sets in a general CRS. This algorithm has a worst-case running time of *O*(|*R*|^2^ log|*R*|), i.e., it is efficient (polynomial in the size of the full reaction network). In previous work, we have applied the RAF algorithm to random reaction networks with up to several millions of reactions, on which the average running time was sub-quadratic [25].

A CRS may not contain any RAF, but when it does it always contains a unique *maximal* RAF (maxRAF), and this maxRAF is the one the RAF algorithm finds. Moreover, it has been shown that a maxRAF can often be decomposed into several smaller subsets which themselves are RAF sets (subRAFs) [28]. If such a subRAF cannot be reduced any further without losing the RAF property, it is referred to as an *irreducible* RAF (irrRAF). The existence of multiple autocatalytic subsets can actually give rise to an evolutionary process [5], and the emergence of larger and larger autocatalytic sets over time [28]. In this paper we will also consider a more restrictive type of autocatalytic set called a “constructible” autocatalytic set (CAF set) [29]. This is an RAF set can be dynamically realized without any of its reactions having to happen “spontaneously” (uncatalyzed) initially to get all catalysts present in the system.

**Figure 1:**
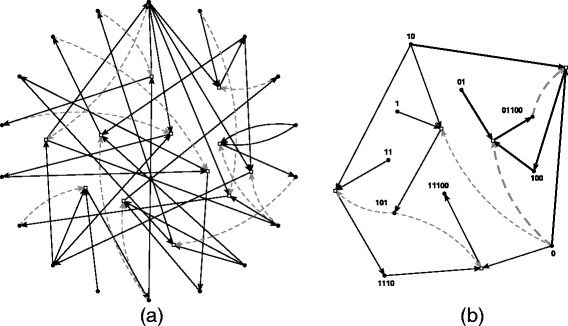
**An example CRS and its (sub)RAFs.** **(a)** An instance of the binary polymer model (catalyzed reactions only). Black dots (on the outside, around a circle) indicate molecule types (not labeled), and white boxes (inside the circle) indicate reactions. Solid black arrows indicate molecules going into and coming out of reactions, and dashed grey arrows indicate catalysis. **(b)** The maxRAF as found by applying the RAF algorithm to the CRS in (a). This maxRAF contains an irreducible RAF of two reactions (indicated by the bold arrows). The food set consists of the bit strings of length one and two.

A simple example of a CRS and its RAF (sub)sets, using the well-known binary polymer model, is given in Figure 1. In the binary polymer model [3,4], molecules are represented by bit strings up to a given length *n*, and the possible reactions are ligation and cleavage. In a ligation reaction, two bit strings are “glued” together into a longer bit string, e.g., 00+111→00111. In a cleavage reaction, a bit string is “cut” into two smaller substrings, e.g., 101010→1010+10. Finally, the bit strings are assigned randomly as catalysts to the possible reactions according to a given probability of catalysis *p* (which is the probability that an arbitrary bit string catalyzes an arbitrary reaction).

Figure 1(a) shows an instance of the binary polymer model with *n*=5, *p*=0.0045, and a food set consisting of all bit strings of length one and two. Only the catalyzed reactions (12 in total) are shown. Black dots indicate molecule types (not labeled), and white boxes indicate reactions. Solid black arrows indicate molecules going into and coming out of reactions, and dashed grey arrows indicate catalysis. Applying the RAF algorithm to this CRS results in the maxRAF shown in Figure 1(b), which consists of five reactions. Furthermore, the maxRAF contains an irreducible RAF of two reactions (indicated by the bold arrows). Note that neither the maxRAF nor this irrRAF is a CAF, as one of the two reactions in the irrRAF needs to happen spontaneously initially before the full RAF set can be realized dynamically.

Using the binary polymer model, it was shown that RAF sets are highly likely to exist, even for very moderate levels of catalysis (between one and two reactions catalyzed per molecule, on average) [25,26,29,30]. Moreover, this result still holds under various model extensions, such as a more realistic “template-based” form of catalysis [27,31,32], a power law distribution of catalysis [33], and even non-polymer systems [34].

In [28] it was shown that, in principle, there can be an exponentially large number of irrRAFs within a given maxRAF. So, there is no hope of efficiently enumerating all irrRAFs within an arbitrary RAF set. Furthermore, in [35] it was shown that even finding the *smallest* irrRAF is NP-complete. However, the RAF algorithm can be extended to provide a method to randomly sample irrRAFs from a given RAF set ${\mathcal {R}}'$, as follows [35]:

**irrRAF sampling algorithm**

1. Randomly reorder the list of reactions contained in ${\mathcal {R}}'$.
2. For each next reaction $r_{i} \in {\mathcal {R}}'$ do:
  - Remove *r*_*i*_ from ${\mathcal {R}}'$.
  - Apply the RAF algorithm to ${\mathcal {R}}'$.
  - If the resulting (maximal) RAF set ${\mathcal {R}}''$ is non-empty, set ${\mathcal {R}}'={\mathcal {R}}''$, otherwise return *r*_*i*_ to ${\mathcal {R}}'$.
3. Return the irreducible RAF set ${\mathcal {R}}'$.

Sampling irrRAFs, and also keeping track of the sizes of the intermediate subRAFs while iterating step 2, can provide useful insight into the modularity of RAF sets, as we show in our results below.

Finally, real autocatalytic sets have actually been constructed in laboratory experiments [6-10]. Recently it was shown that the formal RAF framework can be directly applied to such real chemical systems, not only reproducing the experimental results, but also providing predictions about the system’s behavior that would be difficult to obtain from the chemical experiments alone [36]. However, these examples, although real, have all been carefully designed and created in controlled experiments. Here, we take a first step at applying the formal RAF framework to a biological CRS: the metabolic network of *E. coli*.

### The metabolic network of *E. coli*

All reactions, their functional annotation, and information about the gene product(s) that catalyze them, were retrieved from the most recent *E. coli* metabolic network data set [12]. Each *E. coli* gene was parsed with the UNIPROT information available on 6 March 2013 regarding the type of metals and cofactors [37].

For the purpose of identifying RAF sets within the *E. coli* metabolic network, the following transformations of the metabolic network were performed: i) Transport reactions and reactions localized within the *E. coli* periplasm (except those involved in oxidative phosphorylation) are removed from the network, and the affected molecules are included in the food set. Thus, molecules taken in from the environment (indicated as “xxx[e]” in the original data set) are directly available as food molecules; ii) In reactions catalyzed by a protein that uses a cofactor or metals, these cofactors and metals are assigned as the catalysts for that reaction. This has the effect of integrating organic cofactors into the reaction network. All metals are included in the food set (unless they are organized as FeS clusters, in which case they are treated as synthesized organic cofactors); iii) Reactions catalyzed by a protein that has no known or annotated cofactor are defined as being catalyzed by a general catalyst called “Protein”, which is included in the food set. In case of RNA-dependent reactions, a generic “RNA” catalyst was introduced that also belongs to the food set. This has the effect of keeping cofactor-independent reactions within the set of catalyzed reactions. Moreover, by grouping these reactions with the same generic catalyst (Protein or RNA), we are simplifying the network’s catalyst space without losing biological information. iv) Reactions for which the *E. coli* enzyme is unknown were assigned to another general catalyst called “genCat”, which is also included in the food set, to resolve incomplete data; v) Groups of catalysts with common properties and common biosynthetic pathways, such as menaquinone/ubiquinone, NAD/NADP, or flavins, are grouped together into a “pool” of equivalent catalysts (see Table 1); vi) Bi-directional reactions are split up into two separate reactions, one forward and one reverse, but catalyzed by the same catalyst. This does not affect the network structure in any way, but in some cases makes it more amenable to the RAF analysis; vii) If a reaction requires more than one catalytic molecule and all catalysts need to be present simultaneously, an additional reaction is included that creates a “catalyst compound” from these individual catalysts, which then catalyzes the given reaction. The reaction that creates such a compound is catalyzed by a general catalyst called “X”. These reactions are annotated as Catalyst reactions and do not exist in *E. coli*’s biological metabolic network. This has the effect of allowing several cofactors to be required for a reaction to take place. This transformation is required for the RAF algorithm to work without changing the biological aspect of the network; viii) All reactions where cofactors (e.g., quinones, metals, FAD) were required by a component of the enzyme, whether involved in catalysis or not, have these cofactors in their required catalyst list. This has the effect of making the reactions dependent on molecules like quinones; ix) If a reaction can be independently catalyzed by two or more proteins, all possible pairs “reactions:catalyst” are included, each instance being catalyzed by the cofactors present in the respective protein; x) When the type of metal was not specified, we assumed that any of the divalent-metal ions could catalyze the reaction, and a pool of divalent metal ions is included in the food set. This allows plasticity and mimics biological enzymes.

**Table 1:** **The different catalyst pools**

**Cofactor**	**Abbreviation/Group**
Thiamine pyrophosphate/Thiamin	Thiamin
NAD ^+^/NADH; NADP ^+^/NADPH	nad-pool
Pyridoxal phosphate	pydx5p
Pyridoxal	pydx
Lipoamide	lipoamp
Methylcobalamin/Cobalamin	Cob
Coenzyme A and derivates	coa
Tetrahydrofolic acid and derivates	folate
Menaquinone/Ubiquinone	Q
Pyrroloquinoline quinone	PQQ
Topaquinone	topaquinone
FMN/FMNH _2_ and FAD/FADH _2_ and Riboflavin	Flavins
Glutathione oxidized and reduced	Glutathione
S-Adenosyl methionine	SAM
Siroheme	sheme
Heme B/Heme O	Heme
Heme D	HemeD
All tRNA	RNA
Molybdopterin	Molybdopterin
4Fe-4S; 2Fe-2S; 3Fe-4S	Iron-Sulfur-cluster
Divalent-cations	Divalent-cations

Finally, xi) reactions annotated in [12] as uncatalyzed were assigned a general catalyst called “spont” (included in the food set). We thus assume that these reactions still happen at a high enough rate to be considered relevant. This approximation might seem counter intuitive since in the strict formalism of RAFs, uncatalyzed reactions are outlawed. However, within a cell, several uncatalyzed reactions do occur, at rates high enough not to impair its metabolic function. Prevailing uncatalyzed reactions within autocatalytic sets tend to give rise to molecules that are not members of the set itself but can be incorporated into it, allowing the evolution of new autocatalytic sets [4,38]. Thus, uncatalyzed biological reactions are processes that introduce chemical species (or increase their availability) in the cell’s environment. In practical terms, and since the generic catalysts are part of the food set, including spontaneous reactions in the network by the insertion of the generic catalyst “spont“ is similar to the introduction of the products of these reactions in the food set itself, as long as their substrates are available. In reality, and excluding the cases were energy coupling exists (e.g. electron bifurcation [39] or Q-cycle [40]) catalyzed reactions are no more than spontaneous uncatalyzed reactions whose activation energy is lowered by the action of a catalyst. In the scenario of early evolution, the primordial reactions would have occurred spontaneously with the help of metal ions and simple abiotic cofactors, with protein dependent catalytic reactions appearing later as add-ons [41]. However, the removal of this generic catalyst does not have high impact on the size of the *E. coli* metabolic RAF (see below).

The initial food set thus consists of all molecules exchanged with the environment. This includes the 324 molecule types labeled as “xxx[e]” in the original data set, plus the ones produced in the periplasm, the introduced general catalysts, and a few essential species such as ATP, to make a total of 438 food molecules. The resulting reaction network, consists of 1199 distinct molecules types (including the 438 food molecules), 1826 reactions (belonging to 33 different functional categories) and 42 catalysts. We then applied the RAF framework to analyze this CRS for the existence and structure of autocatalytic sets.

A second food set was created based on the laboratory conditions given for *E. coli* growth on glucose-6-phosphate and glucose as in [42]. The reactions included in the resulting RAF network were mapped to *E. coli* Kegg pathways [43]. Hierarchical clustering and plotting of this network were performed in MATLAB.

## Results and discussion

### RAF sets in the metabolic network of *E. coli*

Applying the RAF algorithm to the *E. coli* metabolic network results in a maximal RAF set (maxRAF) consisting of 1787 reactions. This corresponds to 98% of the full 1826-reaction metabolic network, that is, only 39 reactions of the full network are not part of the maxRAF. When ATP or an equivalent compound such as ADP is available in this food set, the resulting RAF set is also a so-called “constructible” autocatalytic set (CAF set). This is in agreement with previous results from [11] where ATP was identified as an obligate autocatalytic metabolite in all metabolic networks studied.

An outline of the number of reactions per functional category in the maxRAF is presented in Table 2, where it can be seen that all the major functional categories, such as amino acid biosynthesis, carbon metabolism, and cofactor and prosthetic group biosynthesis, are represented within the RAF set.

**Table 2:** **The number of reactions in the maxRAF set belonging to each functional category**

**Functional category**	**Reactions**
Alanine and Aspartate Metabolism	11
Alternate Carbon Metabolism	217
Anaplerotic Reactions	11
Arginine and Proline Metabolism	45
Cell Envelope Biosynthesis	135
Citric Acid Cycle	23
Cofactor and Prosthetic Group Biosynthesis	235
Cysteine Metabolism	13
Folate Metabolism	11
Glutamate Metabolism	6
Glycerophospholipid Metabolism	150
Glycine and Serine Metabolism	17
Glycolysis/Gluconeogenesis	34
Glyoxylate Metabolism	4
Histidine Metabolism	12
Inorganic Ion Metabolism	32
Lipopolysaccharide Biosynthesis / Recycling	39
Membrane Lipid Metabolism	78
Methionine Metabolism	16
Methylglyoxal Metabolism	10
Murein Recycling	20
Nitrogen Metabolism	13
Nucleotide Salvage Pathway	173
Oxidative Phosphorylation	65
Pentose Phosphate Pathway	19
Purine and Pyrimidine Biosynthesis	35
Pyruvate Metabolism	23
Threonine and Lysine Metabolism	25
Tyrosine, Tryptophan, and Phenylalanine Metabolism	29
Unassigned	21
Valine, Leucine, and Isoleucine Metabolism	23
tRNA Charging	23
Catalysts Reaction	219
**Total**	**1787**

### Minimum food set

A crucial determinant for the existence (and size) of RAF sets is the composition of the food set. As described above, the initial food set for the *E. coli* metabolic network consists of 438 molecule types, 324 of these being chemical species that are exchanged with the environment in [12]. A large redundancy is found within this last group, namely in terms of redox state of the uptaken metals or interconvertible chemical pairs (e.g. N-acetyl-D-galactosamine and N-acetyl-D-galactosamine 1-phosphate). Nevertheless, they provide a good starting point for this analysis. The additional molecules of the food set are those produced in the periplasm, the introduced general catalysts, and a few essential molecules such as ATP. Although ATP is produced by the reaction system, it needs to be part of the food set, because otherwise the reaction system does not move forward initially. This role of ATP in biological networks has already been pointed out in [11] where it was shown that regardless of the initial food set, ATP or equivalent compounds participating in the same autocatalytic cycle are obligatory autocatalytic metabolites. In the context of early evolution, this can be seen as a requirement for favorable thermodynamics in spontaneous chemical evolution [44-46].

However, this initial food set can be reduced without diminishing the size of the maximal RAF set. Out of the 438 initial food molecules, there are 117 “essential” ones: removing any one of these individually reduces the size of the maximal RAF set. In the majority of cases (103) the RAF set is reduced by less than 30 reactions, but there are 7 molecules that reduce the size of the RAF set by more than 1000 reactions when removed from the food set, in particular the generic catalysts.

Removing the remaining 321 molecules from the food set, i.e., using only the 117 essential food molecules, also results in a reduced maxRAF. So, at least some subset of these 321 molecules needs to remain in the food set to maintain the maxRAF. This brings up the question of whether it is possible to (efficiently) find a minimum food set that maintains a given RAF set in a reaction network. Unfortunately, this problem turns out to be NP-complete, as the following theorem states.

Consider the following combinatorial optimization problems.

**min-F RAF:**

ᅟ INSTANCE: A CRS ${\mathcal {Q}}=(X,{\mathcal {R}},C)$, and food set *F*⊆*X*, with ${\mathcal {R}}^{\prime } \subseteq {\mathcal {R}}$ an RAF for $({\mathcal {Q}}, F)$, and a positive integer *k*.
ᅟ QUESTION: Is there a subset *F*^′^ of *F* of size at most *k* for which ${\mathcal {R}}^{\prime }$ is an RAF for $({\mathcal {Q}}, F^{\prime })$?

**min-F generation:**

ᅟ INSTANCE: A set of reactions ${\mathcal {R}}^{\prime }$ that is *F*−generated, and a positive integer *k*.
ᅟ QUESTION: Is there a subset *F*^′^ of *F* of size at most *k* for which ${\mathcal {R}}^{\prime }$ is *F*^′^−generated?

Recall that *F*−generated means that each reactant in ${\mathcal {R}}^{\prime }$ is either present in *F* or can be created from *F* by using a series of reactions only from ${\mathcal {R}}^{\prime }$ itself.

#### Theorem 1.

The min-F RAF and min-F generation problems are NP-complete.

#### *Proof*.

See the Appendix.

This seems a rather technical result, but it implies that there is no hope of constructing an efficient (polynomial-time) algorithm for finding a minimum food set to maintain the same maxRAF set. This, therefore, presents a limit to our ability to study a given reaction network analytically.

However, we can still take a heuristic approach and construct a randomized search algorithm to sample food subsets, and then take the smallest set from the resulting samples as an approximate solution to the min-F problem. This randomized algorithm is similar to that for sampling irreducible RAF sets as described in section “Autocatalytic sets”, and analogous to the method used in [47] to find minimal metabolic networks.

**min-F search algorithm**

1. Randomly reorder the list of molecule types the original food set *F*.
2. For each next element *f*_*i*_∈*F* do:
  - Remove *f*_*i*_ from *F*.
  - Apply the RAF algorithm.
  - If the resulting (maximal) RAF set is smaller than before, return *f*_*i*_ to *F*, otherwise leave it out.
3. Return the (reduced) food set *F*.

Repeating this algorithm any number of times on the *E. coli* data set always returns the same smallest size for the food set of 123 molecules. This includes the 117 essential molecules, plus some combination of six molecule types from the remaining 321 compounds. This combination of six molecule types is not always the same, though, but they do come from a very small subset. Table 3 shows an example of ten possible combinations found by our randomized search algorithm.

**Table 3:** **Ten different combinations of the additional six food molecules necessary to maintain the maximal RAF set**

**Molecule**	**1**	**2**	**3**	**4**	**5**	**6**	**7**	**8**	**9**	**10**
ATP	x						x			
Cob(I)alamin	x								x	
fructoselysine	x	x		x		x		x	x	
D-Fructuronate	x			x	x			x		
D-Gluconate	x					x		x		
SO _2_	x	x			x	x	x	x		
Adenosylcobalamin		x	x	x	x	x	x	x		x
ADP		x		x				x	x	
D-Glucuronate		x	x			x	x		x	x
L-Idonate		x	x	x	x		x			x
GTP			x		x	x				x
H _2_ *O*_2_			x						x	x
psicoselysine			x		x		x			x
O _2_				x						
5-Dehydro-D-gluconate									x	

Table 3 reveals the presence of groups of molecules such as adocbl (adenosyl-cobalamin) and cbl1 (cobalamin), or atp/adp/gtp. These grouped molecules correspond to equivalent metabolites in the sense that they can break down the same autocatalytic cycle [11] and the presence of any molecule from each of these groups in the food set is sufficient to maintain the original maxRAF. Thus, even though the general problem of finding the minimum food set is NP-complete, in case of the *E. coli* network it seems very likely that the minimum food set is of size 123, but that it is not unique.

With such a minimum food set, however, the maxRAF set is not “constructible” anymore (it would need to have some catalyst-requiring reactions occurring without catalyst to begin with untill all catalysts have been generated), although there is still a CAF subset of 434 reactions within the maximal RAF. For the remainder of our analysis, we use the essential 117 molecules plus the first set of six additional molecules from Table 3 as the (minimum) food set.

This large number of “essential” molecules in the food set is to some extent an artifact arisen from how the initial metabolic network was constructed [12] and from the condition we imposed that the size of the maximal RAF should not be reduced. Most of the essential food molecules correspond to specifications of generic compounds involved in the peripheral glycerophospholipid metabolism (seven derivatives each of 2-acyl-sn-glycero-3-phosphoethanolamine, 2-acyl-sn-glycero-3-phosphoglycerol and enoyl-sn-glycerol 3-phosphate), post-translationally modified amino acids or configurations of usually rare biological isomers that participate in few reactions (five post-translationally modified amino acids, one D-aminoacid, five L-sugars and D- and L-tartaric acid). In all of these cases, no reaction(s) exist for their synthesis in the network although they participate as substrates. For the same reason, additional molecules such as lipoate, dopamine, ferric 2,3-dihydroxybenzoylserine or Fe(III) hydroxamic acid, also need to be included in the food set as essential molecules.

Another factor contributing to the large number of food molecules are environmental adaptations of *E. coli*. As recently summarized by Mackie et al. [42] *E. coli* can grow under different conditions, for example, under aerobic and anaerobic conditions, or using different carbon sources. The extended food set allows for all of these alternative pathways to be functioning within the RAF network. This raises the question of why to use *E. coli* as a model organism for probing questions regarding self-organization of primordial metabolism. The reason is simple: even though other metabolic networks exist [48,49], *E. coli*’s is the best studied and annotated biological network available, even if not perfect (see Conclusions). Finally, five generic catalysts contribute to the large number of food set molecules. These are artificial constructs within the network and both their impact and biological significance within the RAF differ (see below). Additional molecules such as thiamin also needed to be included in the food set as essential molecules, although within the metabolic network, reactions for thiamin synthesis do exist. This apparently counterintuitive measure has two reasons. First, our network does not contain any biosynthetic route for the synthesis of 4-methyl-5-(2-hydroxyethyl)-thiazole, a thiamin precursor involved in the pyrimidine branch of thiamin biosynthesis. Second, even if this molecule is included in the food set, the double autocatalytic nature of the thiazole branch of thiamin biosynthesis in *E. coli* would prevent these reactions to be included in the RAF set. Briefly, the enzymes involved in the biosynthesis of thiamin are dependent, among other cofactors, on PLP [50] and thiamin itself [51]. On the other hand, the biosynthesis of PLP, besides being PLP dependent [52], has one step that is thiamin dependent [51]. Thus, in order to include these reactions within the RAF system, several reactions would have to proceed uncatalyzed or one of the cofactors would have to be present in the food-set. This atributte suggests that at the origin of life, some forms of the cofactors existed, hence were synthesized spontaneously, before their biosynthetic pathway arose. The remaining essential molecules of this food set, consist mainly of 20 different metals, nine inorganic compounds including gases, 28 distinct carbon and sulphur sources, and ATP. The full list of this food set is presented in the Additional file 1.

### The role of catalysts

Out of the 1199 molecule types in the network, only 42 (3.5%) act as catalysts, either by themselves or as part of a catalyst compound. Thus, in the *E. coli* metabolic network, most molecules do not catalyze any reactions at all, some catalyze a few reactions, and there are a few molecules that catalyze many reactions.

Table 4 lists all 42 catalysts, ordered by the number of reactions they catalyze (“cat”; these include reactions they catalyze as part of a composite catalyst). The table also shows by how many reactions the maxRAF is reduced if each of these 42 catalysts is removed individually from the network (labeled as “rem”). As expected, the generic catalysts “Protein” and “X” affect the RAF set by reducing its size drastically. This is because of the large number of reactions they catalyze (as with “Protein”), or because all reactions catalyzed by them produce other catalysts or groups of catalysts essential for other reactions in the RAF set (as with “X”). In the current *E. coli* RAF network, at least 65% of the reactions are catalyzed by a cofactor and this number would be larger were complete annotations for the genes available (see Conclusions). So, even with a possible bias toward Protein catalyzed reactions, that participate and/or connect different metabolic pathways, we still recall the importance of cofactors within this metabolism. In contrast, the removal of the generic catalyst introduced to allow for uncatalyzed reactions (spont) does not have a significant impact on the size of the *E. coli* metabolic RAF.

**Table 4:** **The 42 catalysts ordered by the number of reactions they catalyze (cat), also indicating by how many reactions the RAF set is reduced when each catalyst is removed from the network (rem)**

	**cat**	**rem**		**cat**	**rem**
Protein	582	1644	pyruvate	9	630
nad-pool	298	1353	Calcium	9	31
X	185	1642	Cob	6	15
Magnesium	142	1476	Copper	5	31
flavin	139	1353	Nickel	5	11
coa	103	619	sheme	5	7
pydx5p	90	1353	lipoamp	4	8
Iron-Sulfur-cluster	86	1353	Potassium	3	166
Zinc	81	1432	topaquinone	3	5
Divalent-cations	78	1413	Molybdenium	10	18
Q	60	98	pan4p	2	625
Iron	55	1353	dpcoa	2	621
Manganese	29	145	Glutathione	2	30
Molybdopterin	28	67	Tungsten	2	11
SAM	27	159	hemeD	2	5
folate	24	682	PQQ	2	5
genCat	23	1367	pydx	2	4
RNA	21	96	prpp	1	1377
heme	18	31	HCO _3_	1	174
Thiamin	17	1378	Sodium	1	4
spont	17	42	Chloride	1	2

When some catalysts that participate in only a few reactions are removed from the network, a large decrease in the size of the RAF can be observed. For example, 5-phosphoribosyl diphosphate (PRPP) is only a catalyst in the conversion of uracil to UMP (and is a substrate in only 13 reactions), but the removal of this conversion reaction reduces the RAF size by 1377 reactions, even though UMP can be produced by seven other distinct reactions. This shows the central role of PRPP in biological networks. In *E. coli*, PRPP is involved in many metabolic pathways being the precursor of histidine and NADH. Within this food set, the only possible route for the *de novo* synthesis of NADH depends on a reaction where PRPP reacts with quinolinate to form nicotinate D-ribonucleotide. Thus, without PRPP there is no NADH synthesis and the network falls apart.

**Figure 2:**
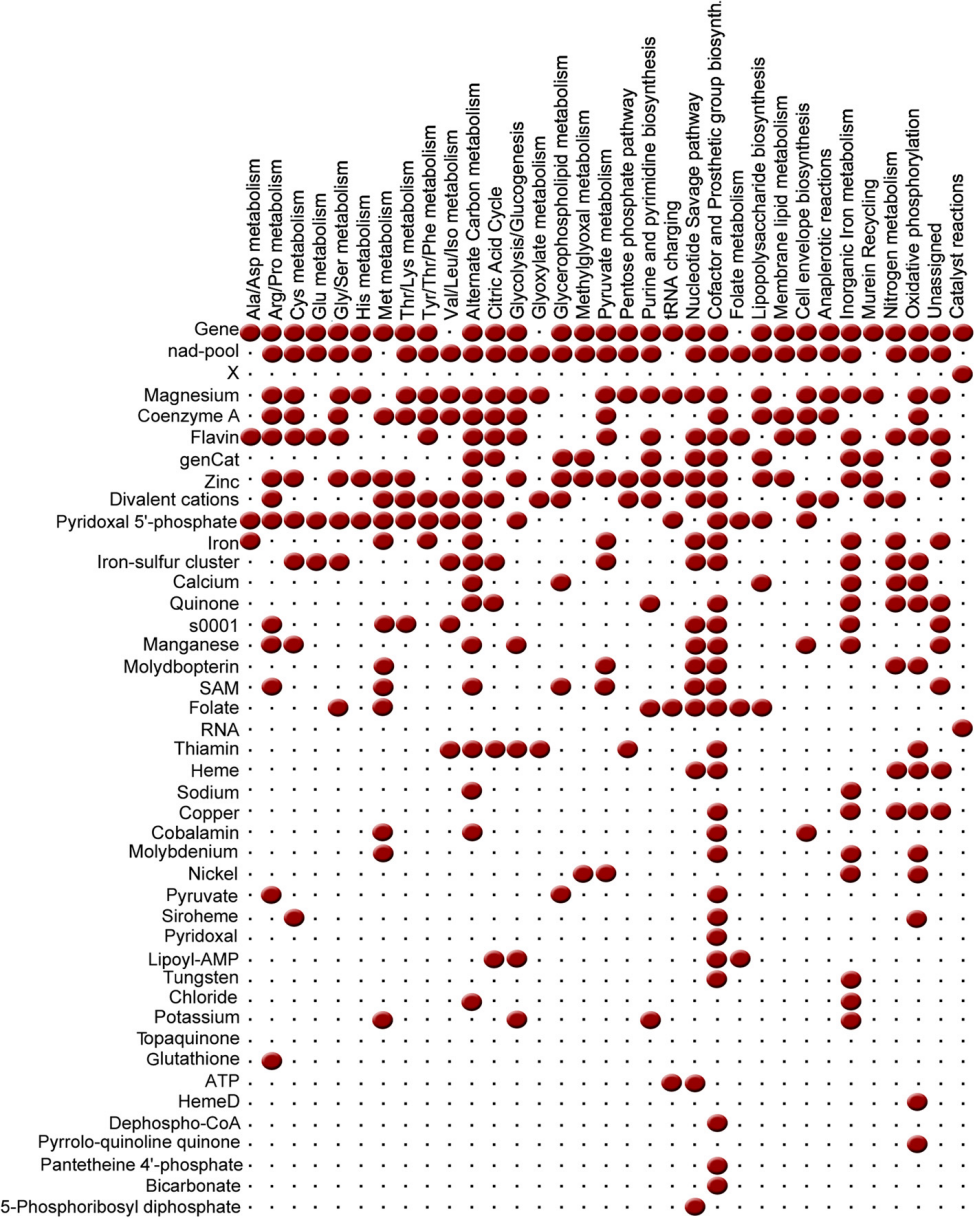
**Distribution of the catalysts over the different functional categories.** The participation of each one of the catalysts (rows) over the different functional categories (columns) is represented by a red dot.

The extensive biological action of the different cofactors can be observed by the uneven distribution of the catalysts according to their functional annotations, see Figure 2.

### Modularity in the RAF set

The maxRAF contains 98% of the reactions in the *E. coli* metabolic network. An obvious question arises: “Is there any modularity in this RAF set?”

First, we looked at hierarchical levels of reactions. Starting with the full maxRAF and only the food molecules, we considered all reactions that can proceed catalyzed, i.e., all reactions in the maxRAF that have all their reactants and at least one of their catalysts in the molecule set. This represents the “level 0” reactions. Next we added all the products of these level 0 reactions to the molecule set, and considered all additional reactions that then proceed catalyzed. This represents level 1 (all reactions one reaction step away from the food set). We repeated this procedure for subsequent levels, until the number of reactions that can proceed catalyzed does not further increase. In short, a reaction in level *i* is *i* reaction steps away from the food set. Since the maxRAF (with the minimum food set) is not a CAF, obviously there will be some reactions that are not in any of these levels. These are the “non-CAF” reactions.

Applying this analysis to the *E. coli* maxRAF results in a number of reactions in each level as shown in Table 5. The reactions in levels 0 to 14, taken together, constitute the 434-reaction CAF subset that exists within the maxRAF (as mentioned above). However, most reactions are in the non-CAF level, i.e., at least some of them will need to happen spontaneously (uncatalyzed) at least once before the full maxRAF can come into existence in a dynamical sense (i.e. before all catalyst are present in the system).

**Table 5:** **The number of reactions in each hierarchical level in the maxRAF of** ***E. coli***

level:	0	1	2	3	4	5	6	7	
reacs:	63	82	102	77	49	16	16	11	
level:	8	9	10	11	12	13	14	non-CAF	
reacs:	7	5	2	1	1	1	1	1353	

Finding a minimum subset of reactions that, when allowed to proceed uncatalyzed at least once, realizes the full maxRAF turns out to be an NP-hard problem [32] (in fact, even just finding the *size* of such a smallest subset is NP-hard). Therefore, determining the distance (number of reaction steps) from the food set of these non-CAF reactions appears to be intractable.

Another way of looking for modularity in an RAF set is as follows. First, construct a “connectivity graph” *G* were each node in *G* corresponds to a reaction in the maxRAF. Next, link reaction *r*_*i*_ to reaction *r*_*j*_ if a product of *r*_*i*_ is either a reactant or a catalyst of *r*_*j*_ transforming *G* in a directed graph. The *strongly connected components* of this digraph can now be computed.

Constructing *G* and computing its strongly connected components for the RAF set in the *E. coli* metabolic network results in 93 such components. However, 87 of these are of size one, five are of size two, and one is of size 1690. This indicates that the maxRAF mostly consists of one large connected component showing, as previously pointed out by [11,13], the auto- and cross-catalytic behavior of metabolic networks.

### The effect of removing reactions

Recall that an irreducible RAF set (irrRAF) is an RAF set from which no reactions can be removed without losing the RAF property. Applying the irrRAF sampling algorithm (as described in section “Autocatalytic sets”) to the maxRAF in the *E. coli* metabolic network (using the minimum food set) always results in an irrRAF of size one. This is not surprising, as our hierarchical levels analysis above resulted in 63 reactions in level 0. Since all of these reactions have their reactants and at least one catalyst in the food set, they are by definition RAF sets by themselves (and, consequently, also irrRAFs, as they are of size one). We refer to these as “trivial” (irr)RAFs. Removing these trivial irrRAFs from the network, however, breaks the original maxRAF, as the resulting RAF set now contains only 17 reactions (with irrRAFs of sizes two and three). So, the “trivial” RAFs are also “essential” for the full maxRAF.

**Figure 3:**
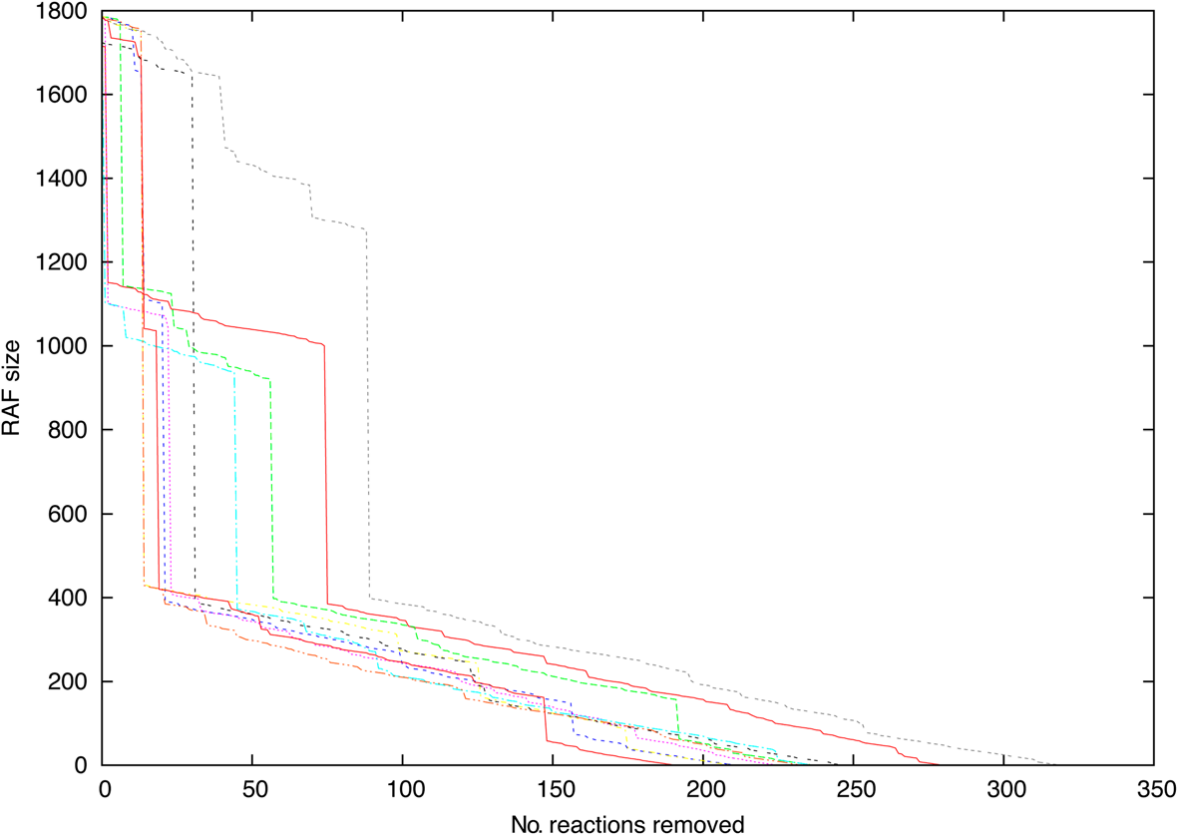
**Ten sequences of repeatedly and randomly removing reactions.** Thin lines represent the impact of randomly removing a reaction in the RAF size. Each line decrease correspond to the removal of a single reaction from the network.

**Figure 4:**
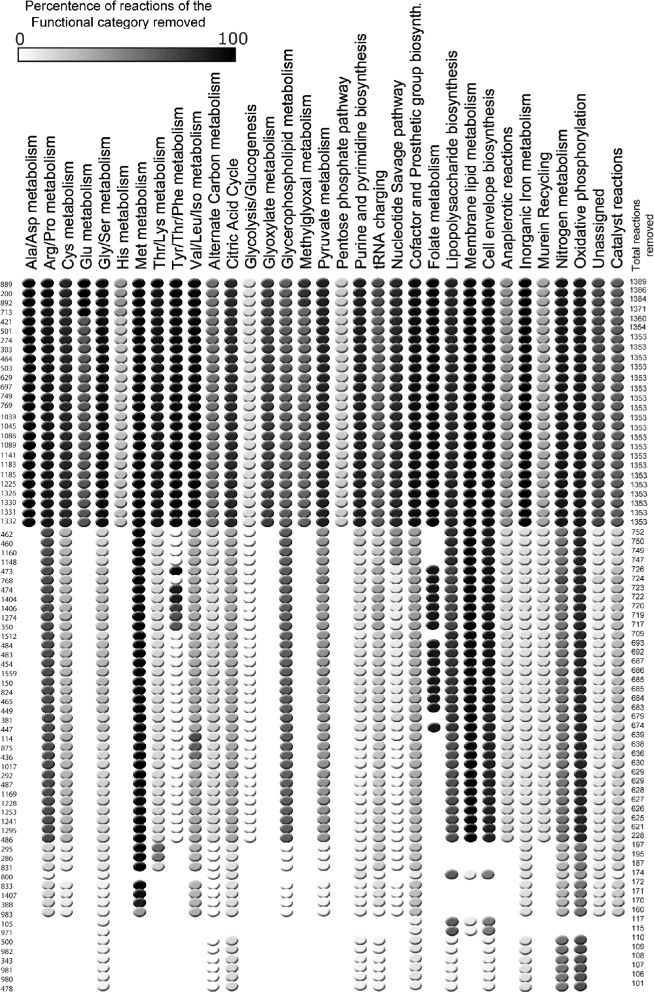
**Functional effect of the removed reactions that decrease the RAF-size by more than 100.** There were 13 reactions involving the synthesis of composite cofactors that reduced the RAF size by more than 100 reactions that are not shown in the Figure. These reactions often comprised the coupling of, for example, folate and magnesium, or thiamine and magnesium, i.e., reactions in which more than one cofactor was required. They were removed from the list so that only *E. coli* reactions and not those generated by recoding of the data are represented. An additional seven reactions in the list that resulted solely from the use of different designations for the same compound in the *E. coli* metabolic network and the *E. coli* Uniprot database were also excluded.

It is still informative, however, to apply the irrRAF sampling algorithm and see how the size of the RAF set is reduced with each (potential) removal of a reaction. Figure 3 shows the sizes of the intermediate RAF sets while iterating the sampling algorithm, for ten repetitions of the method. In most cases, the removal of a reaction reduces the RAF size by only a very small amount and only in few cases this removal results in a significant reduction in the RAF size. For each reaction that reduces the RAF size by more than 100 reactions, the functional categories affected are given in Figure 4. Table 6 lists the actual reactions themselves, including their function category. The same pattern shows up for multiple repetitions of the algorithm. However, for readability of Figure 3 we have only shown the results of ten such runs.

**Table 6:** **Functional categories of the reactions affecting RAF size by more than 100 (“decr”)**

**ID**	**Functional annotation**	**Reaction**	**Decr**
889	Murein Recycling	LalaDgluMdap + h _2_o → 26dap-M + LalaDglu	1389
200	Murein Recycling	LalaDglu → LalaLglu	1386
892	Murein Recycling	LalaLglu + h _2_o → ala-L + glu-L	1384
713	Glutamate Metabolism	atp + glu-L + nh _4_ → adp + pi + h + gln-L	1371
421	Cofactor and Prosthetic Group Biosynthesis	ru5p-D → db4p + h + for	1360
501	Cofactor and Prosthetic Group Biosynthesis	g3p + h + pyr → co _2_ + dxyl5p	1354
274	Cofactor and Prosthetic Group Biosynthesis	5apru + h + nadph → 5aprbu + nadp	1353
303	Alanine and Aspartate Metabolism	glu-L + oaa → akg + asp-L	1353
464	Cofactor and Prosthetic Group Biosynthesis	25drapp + h + h _2_o → 5apru + nh _4_	1353
503	Cofactor and Prosthetic Group Biosynthesis	e4p + h _2_o + nad → 4per + h + h + nadh	1353
629	Cofactor and Prosthetic Group Biosynthesis	atp + fmn + h → fad + ppi	1353
697	Nucleotide Salvage Pathway	atp + gmp → adp + gdp	1353
749	Purine and Pyrimidine Biosynthesis	atp + gln-L + h _2_o + xmp → amp + ppi + h + h + gmp + glu-L	1353
769	Cofactor and Prosthetic Group Biosynthesis	gtp + h _2_o + h _2_o + h _2_o → 25drapp + ppi + h + h + for	1353
1039	Cofactor and Prosthetic Group Biosynthesis	atp + nad → adp + nadp + h	1353
1045	Cofactor and Prosthetic Group Biosynthesis	atp + dnad + nh _4_ → amp + ppi + nad + h	1353
1086	Nucleotide Salvage Pathway	atp + h + nicrnt → dnad + ppi	1353
1089	Cofactor and Prosthetic Group Biosynthesis	h + h + prpp + quln → co _2_ + ppi + nicrnt	1353
1141	Cofactor and Prosthetic Group Biosynthesis	glu-L + ohpb → akg + phthr	1353
1183	Cofactor and Prosthetic Group Biosynthesis	dxyl5p + nad + phthr → co _2_ + pi + pdx5p + nadh + h _2_o + h _2_o + h	1353
1185	Cofactor and Prosthetic Group Biosynthesis	4per + nad → h + nadh + ohpb	1353
1225	Cofactor and Prosthetic Group Biosynthesis	5aprbu + h _2_o → 4r5au + pi	1353
1326	Cofactor and Prosthetic Group Biosynthesis	dhap + iasp → h _2_o + quln + pi + h _2_o	1353
1330	Cofactor and Prosthetic Group Biosynthesis	atp + ribflv → adp + h + fmn	1353
1331	Cofactor and Prosthetic Group Biosynthesis	4r5au + db4p → dmlz + pi + h _2_o + h _2_o	1353
1332	Cofactor and Prosthetic Group Biosynthesis	dmlz + dmlz → 4r5au + ribflv	1353
462	Purine and Pyrimidine Biosynthesis	cbasp + h → dhor-S + h _2_o	752
460	Purine and Pyrimidine Biosynthesis	dhor-S + fum → orot + succ	750
1160	Purine and Pyrimidine Biosynthesis	orot + prpp → orot5p + ppi	749
1148	Purine and Pyrimidine Biosynthesis	h + orot5p → co _2_ + ump	747
473	Tyrosine, Tryptophan, and Phenylalanine Metabolism	2dda7p → 3dhq + pi	726
768	Cofactor and Prosthetic Group Biosynthesis	gtp + h _2_o → ahdt + h + for	724
474	Tyrosine, Tryptophan, and Phenylalanine Metabolism	3dhq → 3dhsk + h _2_o	723
1404	Tyrosine, Tryptophan, and Phenylalanine Metabolism	3dhsk + h + nadph → nadp + skm	722
1406	Tyrosine, Tryptophan, and Phenylalanine Metabolism	atp + skm → adp + skm5p + h	720
1274	Tyrosine, Tryptophan, and Phenylalanine Metabolism	pep + skm5p → 3psme + pi	719
350	Tyrosine, Tryptophan, and Phenylalanine Metabolism	3psme → chor + pi	717
1512	Nucleotide Salvage Pathway	atp + ump → adp + udp	709
484	Cofactor and Prosthetic Group Biosynthesis	ahdt + h _2_o → dhpmp + ppi + h	693
483	Cofactor and Prosthetic Group Biosynthesis	dhpmp + h _2_o → dhnpt + pi	692
454	Cofactor and Prosthetic Group Biosynthesis	dhnpt → 6hmhpt + gcald	687
1559	Cofactor and Prosthetic Group Biosynthesis	chor + gln-L → 4adcho + glu-L	686
150	Cofactor and Prosthetic Group Biosynthesis	4adcho → 4abz + pyr + h	685
824	Cofactor and Prosthetic Group Biosynthesis	6hmhpt + atp → 6hmhptpp + h + amp	685
465	Cofactor and Prosthetic Group Biosynthesis	4abz + 6hmhptpp → dhpt + ppi	684
449	Cofactor and Prosthetic Group Biosynthesis	atp + dhpt + glu-L → adp + pi + h + dhf	683
381	Purine and Pyrimidine Biosynthesis	atp + gln-L + h _2_o + utp → adp + pi + h + h + glu-L + ctp	679
447	Cofactor and Prosthetic Group Biosynthesis	dhf + h + nadph → nadp + thf	674
114	Valine, Leucine, and Isoleucine Metabolism	h + pyr + pyr → alac-S + co _2_	639
875	Valine, Leucine, and Isoleucine Metabolism	alac-S + h + nadph → 23dhmb + nadp	638
436	Valine, Leucine, and Isoleucine Metabolism	23dhmb → 3mob + h _2_o	636
1017	Cofactor and Prosthetic Group Biosynthesis	3mob + h _2_o + mlthf → 2dhp + thf	630
292	Cofactor and Prosthetic Group Biosynthesis	asp-L + h → ala-B + co _2_	629
487	Cofactor and Prosthetic Group Biosynthesis	2dhp + h + nadph → nadp + pant-R	629
1169	Cofactor and Prosthetic Group Biosynthesis	ala-B + atp + pant-R → amp + ppi + pnto-R + h	628
1228	Cofactor and Prosthetic Group Biosynthesis	atp + pnto-R → 4ppan + h + adp	627
1253	Cofactor and Prosthetic Group Biosynthesis	4ppan + ctp + cys-L → 4ppcys + ppi + h + cmp	626
1241	Cofactor and Prosthetic Group Biosynthesis	4ppcys + h → co _2_ + pan4p	625
1295	Cofactor and Prosthetic Group Biosynthesis	atp + h + pan4p → dpcoa + ppi	621
486	Cofactor and Prosthetic Group Biosynthesis	atp + dpcoa → adp + h + coa	619
295	Threonine and Lysine Metabolism	asp-L + atp → 4pasp + adp	197
286	Threonine and Lysine Metabolism	4pasp + h + nadph → aspsa + nadp + pi	195
831	Threonine and Lysine Metabolism	aspsa + h + nadph → hom-L + nadp	187
800	Unassigned	co _2_ + h _2_o → h + hco _3_	174
833	Methionine Metabolism	hom-L + succoa → coa + suchms	172
1407	Methionine Metabolism	cys-L + suchms → cyst-L + succ + h	171
388	Methionine Metabolism	cyst-L + h _2_o → hcys-L + pyr + nh _4_	170
983	Methionine Metabolism	atp + h _2_o + met-L → amet + ppi + pi	160
105	Membrane Lipid Metabolism	accoa + atp + hco _3_ → adp + pi + malcoa + h	117
971	Membrane Lipid Metabolism	ACP + malcoa → coa + malACP	115
500	Cofactor and Prosthetic Group Biosynthesis	dxyl5p + h + nadph → 2me4p + nadp	110
982	Cofactor and Prosthetic Group Biosynthesis	2me4p + ctp + h → 4c2me + ppi	109
343	Cofactor and Prosthetic Group Biosynthesis	4c2me + atp → 2p4c2me + h + adp	108
981	Cofactor and Prosthetic Group Biosynthesis	2p4c2me → 2mecdp + cmp	107
980	Cofactor and Prosthetic Group Biosynthesis	2mecdp + flxr + flxr + h → flxso + h _2_o + h2mb4p + flxso	106
478	Cofactor and Prosthetic Group Biosynthesis	dmpp + ipdp → grdp + ppi	101

Chemical species are symbolic represented.

The reactions whose removal reduces the size of the RAF the most involve nitrogen assimilation, glutamine synthase (GS) and mureine recycling. The latter seems surprising but is easily explained, because in the *E. coli* network we use here [12], a mureine degradation product (anhgm4p, N-acetyl-D-glucosamine N-acetylmuramyl-tetrapeptide), originally a periplasmic product but transferred to the food set (see Experimental), is one of the food sources of glutamate, the substrate for ammonium incorporation in the GS reaction. Within this RAF network, the highly dependent cofactor biosynthesis network is initially only operational at the expenses of glutamate formation from the mureine degradation pathway. If glutamate (or GS) is removed, no nitrogen can be incorporated and the reaction network stalls at many reactions, much like the case of ATP above. Glutamine synthase is the entry point of nitrogen in *E. coli* metabolism [53], and without nitrogen, no cofactors, amino acids or bases can be generated, so this result makes sense.

Also of interest is the central role that cofactor biosynthesis assumes in the *E. coli* RAF. Of the 76 reactions that reduce the size by more that 100 reactions, 43 (57%) fall in the functional category synthesis of cofactor and prosthetic groups. This is intuitively understandable because cofactors are catalysts. It furthermore underscores the importance of cofactors as mediators of metabolism [11,54,55]. Following the cofactors in terms of effect on the RAF when removed, are amino acids (21), nucleotide biosynthesis (9), and three others.

Finally, there are clear intermediate levels in the (reducing) RAF sets. As Figure 3 shows, there are significant drops in RAF size first to around 1100 reactions and then to around 400 reactions, which occur in almost all of the 10 runs. Interestingly, all these (roughly) size-400 subsets consist primarily of reactions from the 434-reaction CAF subset, indicating that this is kinf of “robust” core of the maxRAF and furthermore indicating the existence of modularity in the RAF set.

### The effects of removing molecules

**Figure 5:**
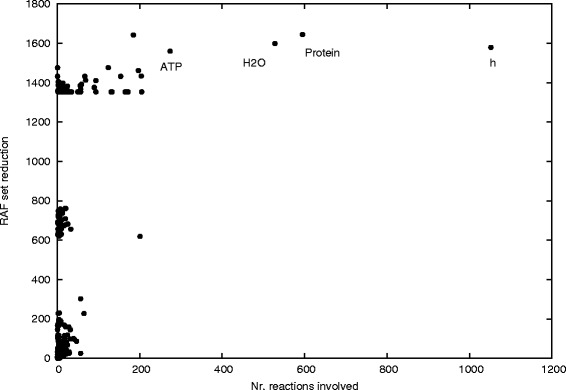
**Involvement and importance of molecule types.** Relationship between the removal of each molecule and the number of reaction where it participates. Each dot corresponds to a different molecule.

The irrRAF search algorithm gives insight into the importance of individual reactions on the size of the RAF set. We can perform a similar procedure for the molecules. Figure 5 shows the reduction in the size of the maxRAF set (in number of reactions) when an individual molecule type is removed from the network (its “importance”), against the number of reactions this molecule is involved in either as a reactant, product, or catalyst (its “involvement”), for all 1199 molecule types. Note that, as with removing reactions, there is a clear separation into a few different levels of RAF sizes when removing molecules.

Obviously the molecules that are involved in many reactions (the upper set of points in Figure 5) have a large importance, that is, they reduce the RAF set by more than 1300 reactions when removed. There are 76 compounds in this group, including all of the cofactors except folate and coenzyme A, many amino acids and core carbon coumponds like ribose-5-phosphate or phosphoenolpyruvate (Table 6). Removal of such compounds should have a large effect. However, there are also many molecules that have a very low involvement (less than 50 reactions, except coenzyme A) but a very high importance in that they reduce the RAF set by more than 600 reactions when removed. There are 52 compounds in this class, including folate, but mostly central metabolites of core biosynthesis. The third group of molecules encompasses 1071 metabolites with both low involvement and low importance. These might be considered as peripheral in *E. coli* metabolism. But if this is the periphery, it would leave only 128 compounds in the core. This might seem like an unrealistically small core, but we note that there are only 303 essential genes in *E. coli* and the metabolic network model of *E. coli* needs just 250 genes to be able to produce biomass [47] (see Conclusions).

After protons (“h” in Figure 5) and the generic catalyst “Protein”, the most frequent participant in an *E. coli* reaction is water. The involvement of water in many reactions might seem trivial, but water is more central to metabolism than one might think: 70% of the water molecules in the cytosol of exponentially growing aerobic *E. coli* cultures do not stem from the aqueous medium, but they are synthesized through *E. coli* metabolism [56].

### Can real food sets support the RAF structure?

If we set the food set to biologically realistic conditions where *E. coli* has been shown to grow, the RAF network collapses and less than 10% of the reactions are retrieved (Table 7). This apparently negative result shows that the introduction of an additional level of regulation (a reaction only occurs if the reaction catalyst is present in the network) has a massive impact in the way current cells function. As observed for the RAF network with a food set of 123 molecules, the catalysts have a different impact in the size of the RAF (Additional file 1: Table S1). The next step is to check what needs to be further introduced in this reduced food set in order for the essential metabolic pathways such as pentose metabolism, glycolysis, citric cycle, amino acid and cofactor biosynthesis to become functional. While finding the minimal food set is an NP-complete problem, finding a food set that creates a CAF network comprising those metabolic pathways is possible. In fact, besides the addition of the obligate autocatalytic metabolite ATP, using the glucose-6-phosphate food set with the addition of just seven catalysts (NAD, FAD, PLP, thiamin, CoA, lipoate and some cobinamine form), a CAF network with the same size of the resulting RAF set and containing 1517 reactions is retrieved. Although with this food set only 85% of the initial network is retrieved, the main *E. coli* cytosolic metabolism is still captured (Table 7). Interestingly, the autocatalytic metabolites that Szathmary and coworkers found for the *E.coli* metabolic network grown in minimum media also included CoA, NAD and ATP [11]. The difference between their study and ours is that we also took into consideration the cofactor dependency of the enzyme catalyzing the reactions. Thus, our list includes FAD, PLP and thiamin as autocatalytic metabolites due to the existence of several enzymes that are dependent on these cofactors, it includes cobalamin that *E. coli* naturally uptakes from the environment and also lipoate, whose synthesis is not described within the network.

**Table 7:** **Impact of removal of molecules from the “real”** ***E. coli*** **food set in the RAF and CAF size**

**Food molecule**	**RAF size decrease**	**CAF size decrease**
*C* *a* ^2+^	7	7
*Cl*	2	2
*C* *o* ^2+^	33	33
*C* *u* ^2+^	27	27
*K* ^+^	155	360
*M* *g* ^2+^	1117	1117
*M* *n* ^2+^	133	133
${MoO}_{4}^{2-}$	24	24
*N* *a* ^+^	3	3
*N* *i* ^2+^	11	11
*N* *O*_3_	3	3
${SeO}_{4}^{2-}$	6	6
*T* *r* *i* *m* *e* *t* *h* *y* *l* *a* *m* *i* *n* *e* *N*−*o* *x* *i* *d* *e*	2	2
$S_{2}O_{3}^{2-}$	0	0
${WO}_{4}^{2-}$	11	11
*Z* *n* ^2+^	631	631
*ATP*	1206	1206
*O*_2_	47	47
*genCat*	492	492
*Protein*	1352	1352
*RNA*	88	88
*spont*	41	41
*X*	1447	1447
*Glutathione*	30	30
*H*_2_ *O*	0	0
*F* *e* ^2+^	91	91
*N* *H*_4_	0	0
*H*_3_ *P* *O*_4_	0	0
*S* *O*_4_	0	0
*I* *r* *o* *n*−*S* *u* *l* *f* *u* *r*−*c* *l* *u* *s* *t* *e* *r*	0	866
*N* *A* *D*∗	1261	1261
*F* *A* *D*∗	0	1162
*G* *l* *u* *c* *o* *s* *e*−6−*p* *h* *o* *s* *p* *h* *a* *t* *e*	0	0
*P* *y* *r* *i* *d* *o* *x* *a* *l*5^′^−*p* *h* *o* *s* *p* *h* *a* *t* *e*∗	0	673
*C* *o* *A*∗	1099	1099
*H*_2_ *S*	0	194
*T* *h* *i* *a* *m* *i* *n*∗	2	417
*A* *d* *e* *n* *o* *s* *y* *l* *c* *o* *b* *i* *n* *a* *m* *i* *d* *e*∗	10	10
*L* *i* *p* *o* *a* *t* *e*∗	8	8

^*^indicates the 7 added molecules.

However, not all of these 39 food molecules are essential to maintain a similar RAF size (Table 7) and this solution is not unique. For instance, the removal of either SO _4_ or S _2_*O*_3_ has no effect on the size of either the RAF or the CAF because the presence of one can sustain *E. coli*’s growth by providing a sulfur source. A more interesting result comes from the removal of glucose-6-phosphate (the only obvious carbon source) from the food set, since its removal also does not affect the size of the RAF. In this case, the source of carbon becomes ATP itself, by its conversion into adenosine in the nucleotide salvage pathway. This captures an aspect of *E. coli*’s versatility since *E. coli* can grow aerobically or anaerobically, both in silico and in vivo, using adenosine as the sole carbon and nitrogen source [57].

**Figure 6:**
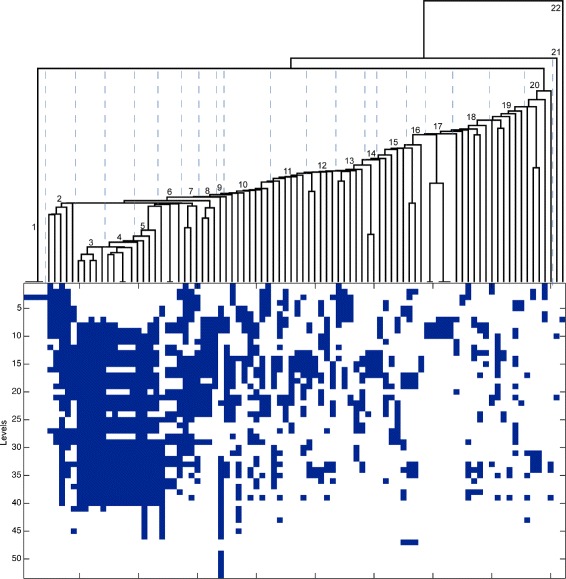
**Hierarchical levels of** ***E. coli*** **CAF network using a “real”? Food set.** Top- Hierarchical clustering dendrogram of *E. coli* metabolic pathways (leafs) according to the hierarchical levels defined in the text. Bottom- Heatmap representing the occurrence of reactions from each one of metabolic pathways (columns) and the 53 hierarchical levels of the CAF network (rows). Blue squares represent the occurrence of at least one reaction from that pathway in a given level. Numbers represent grouped pathways. 1- Nucleotide-excision-, mismatch-, and base-excision- repair; DNA-replication. 2- Glycolysis/gluconeogenesis; methane-, carbon-, amino acids biosynthesis, glycine, serine and threonine- metabolism. 3- Fatty acid biosynthesis and metabolism; valine, leucine and isoleucine-, geraniol- and fatty-acid- degradation. 4 - Lysine degradation; tryptophan metabolism; limonene-, caprolactam- degradation; beta-alanine metabolism. 5- Unsaturated fatty-acids biosynthesis, biotin-, propanoate- and butanoate- metabolism. 6– Glycerophospholipid- and alpha-linolenic acid metabolism; ethylbenzene degradation; pantothenate and CoA biosynthesis. 7- Purine-, pyrimidine- and porphyrin- metabolism; Valine, leucine and isoleucine biosynthesis. 8- Oxocarboxylic acid metabolism; phenylalanine, tyrosine and tryptophan biosynthesis. 9- Lipopolysaccharide biosynthesis. 10- Arginine and proline-, amino sugar and nucleotide sugar-, glycerolipid-, histidine-, glyoxylate- and dicarboxylate metabolism; benzoate degradation; nicotinate-, starch and sucrose metabolism. 11- Quinone biosynthesis; pyruvate-, galactose- metabolism; lysine biosynthesis; PLP metabolism; aminoacyl-tRNA biosynthesis. 12- Cysteine and methionine metabolism; siderophore-group nonribosomal-peptides biosynthesis; glutathione-, citrate-cycle, sulfur- metabolism. 13- Pentose phosphate pathway; fructose-, mannose metabolism; pentose and glucuronate interconversions; peptidoglycan- and folate- biosynthesis. 14- Phenylalanine metabolism; novobiocin biosynthesis. 15– Tyrosine-, riboflavin and cyanoamino-acid metabolism; terpenoid-backbone biosynthesis; Selenocompound metabolism. 16– Thiamine-, Sulfur-relay system, D-Alanine metabolism. 17– Dioxin-, Xylene-, Chloroalkane-, naphthalene-, aromatic- degradation. 18- C5-Branched dibasic acid-, inositolphosphate-, oxidative phosphorylation, nitrogen-, two-component system, taurine- metabolism. 19- lipoic acid-, alanine, aspartate, glutamate-, D-glutamine and D-glutamate- metabolism; nitrotoluene degradation; folate one-carbon pool; ascorbate metabolism. 20- Aminobenzoate degradation; streptomycin-, polyketide-sugar biosynthesis; RNA-, toluene- degradation. 21- Arachidonic acid metabolism. 22- phosphotransferase system.

To check the modularity of this CAF network in terms of the metabolic pathways organization, we determined the hierarchical levels of each reaction, grouped by *E. coli* KEGG metabolic pathways. Based on the relationship between the different *E. coli* metabolic pathways with the 53 hierarchical levels retrieved, it is possible to represent the CAF network in a tree-based structure containing several grouped pathways (Figure 6). In this representation, thr reaction network is organized into 53 different levels of iteration, starting from the 39-molecule food set. Similar patterns of occurrence with increasing distance from the food set indicate that the molecules and catalysts necessary for each of their reactions arise at the same iteration. The synthesis of lipopolysaccharide, the major component of *E. coli* outer membrane (group 9 in Figure 6) is the last process to be concluded suggesting a high dependency of lipopolysaccharide biosynthesis on other metabolic pathways and, consequently, its late emergence during metabolic evolution.

## Conclusions

Although autocatalytic networks are found in biological systems, the systematic impact of including the metal and cofactor protein dependencies as catalysts in metabolic networks under the RAF context has not been previously investigated. The present analyses shows that, within this framework, the *E. coli* metabolic network can indeed be expressed as an RAF set. RAFs also recover the modularity and hierarchical behavior of the *E. coli* metabolic network – in particular they underscore the crucial role of cofactors as the prime mediators of metabolism, a recurring theme in the study of metabolic architecture [11,55,58]. Here we have shown the important role of metals and molecules such as NAD, ATP and CoA in breaking autocatalytic cycles and sustaining the network complexity. This result is in agreement with findings of Heinrich and coworkers [59], who analysed the scopes of compounds and expansion within KEGG metabolic networks and showed the crucial role of the inclusion of these metabolites in the expansion and robustness increase of metabolic networks. Moreover, by also including the metal and cofactor dependencies of the proteins, we extended this set of compounds by identifying additional molecules such as thiamin and PLP as autocatalytic metabolites within *E. coli* metabolism. Thus, RAFs can clearly be used to explore the biological importance of molecules/catalyst within a cell and their interrelationships. But there are caveats.

Among the caveats to the present analyses, the underlying databases are not complete. As one example, biotin does not occur as a cofactor in the present annotations from the UNIPROT database for *E. coli*, but it is known to generally be required in ATP-dependent carboxylation reactions, for example in that catalyzed by acetyl-CoA carboxyase [60]. Another caveat is that RAFs exclude, by definition, a potentially important kind of reaction, namely spontaneous reactions that have no catalysts at all. There are a number of important reactions in biology that are spontaneous. A prime example is the first step in CO _2_ assimilation in methanogens, which involves the spontaneous (non-enzymatic) formation of N-carboxymethanofuran [61]. In modern environments, about a billion tons of carbon are processed via this spontaneous reaction each year [62] and spontaneous reactions of this type might have been important in early evolution [55]. For this reason we introduced the catalyst “spont” for reactions that are annotated as spontaneous, of which there are 17 in the present analysis (Table 4).

Another caveat is promiscuity (or messiness) in enzyme function, that is, the inherent ability of enzymes to catalyze several different selectable reactions [63], whereby usually only one function appears on metabolic maps. This opens the possibility to have additional parallel reactions catalyzed by different cofactors within the metabolic network. Early in the evolution of enzymes, that is at the dawn of protein folds and enzyme families, catalytic specificity was probably rare. In modern *E. coli*, the full extent to which gene products can substitute for each other is not known, although in one classical study, 620 genes in *E. coli* were found to be essential in rich medium (263 of which had no known function), while 3126 were dispensable [64]. A more recent study found that only 303 *E. coli* genes were essential (37 of which had no known function), and 3985 were dispensable [65]. This indicates that there is a great deal of redundancy and/or environmental specificity [66] built into *E. coli* metabolism and that there is still much to be learned about its map.

Finally, although we can easily find RAFs, including CAFs and irreducible RAFs, there are also limits to what can be calculated. For example, we have shown here that finding the minimal food set needed to maintain a given RAF is a computationally intractable (NP-complete) problem in general. So too is finding a smallest irrRAF [35], but here this seems not interesting as the smallest irrRAF turn out to be of size one (so-called “trivial” irrRAFs). Instead, it would be of more interest to find a *largest* irrRAF within the *E. coli* metabolism network. However, at present it is not clear whether this can be computed efficiently.

### Do these findings bear upon early chemical evolution?

The origin and initial interest in RAFs stem from early speculation about chemical evolution [4] and the possibility that autocatalytic sets might have played a role as a means of self organization en route to higher complexity prior to the advent of genetically specified catalysts. One prerequisite for the existence of RAFs in the real world is of course a set of food molecules provided by the environment. Another prerequisite is that the laws of thermodynamics must be obeyed, thus that the overall reaction needs to release energy. Amend et al. [45] have examined these two properties in the context of hydrothermal vents, where organic synthesis from smaller “food” building blocks is thermodynamically favored, owing to the exergonic nature of the interactions between H _2_ and CO _2_ to yield organic products. Of interest, Kauffman’s speculations entailed the synthesis of large peptides from small ones, and early work showed that the synthesis of both amino acids and peptides under hydrothermal vent conditions are exergonic processes [67], whereby a typical microbe is more than 50% protein by weight [68,69].

*E. coli* is a heterotroph that can live anaerobically but can also, like human mitochondria, use O _2_ as the terminal electron acceptor in its ATP-generating electron transport chain. In that sense the *E. coli* metabolic network is hardly an ideal model for early chemical evolution. In addition, during evolution abiotic catalysts and metals in primordial RAF sets have been replaced by sophisticated chemical catalysts, as studies of metal and cofactor gains and losses across protein families have shown [70-72]. Comparative genomic analysis of the distribution of trace elements in current genomes indicate that the loss of a metal or cofactor is more frequent that their respective gain [73]. Moreover, phylogenomic analysis of protein structures also concluded that Fe, Mn, and Mo were preferentially selected by early life forms and were replaced or lost during evolution [74], such that in early chemical evolution, metal dependency was probably higher. Moreover, throughout evolution, protein have often been replaced by analogous proteins of similar or identical functions. The presence across genomes of metal-independent (class I and Ia) and metal-dependent (class II) aldolases is just one example [75-77] where, possibly due to later sugar metabolism adaptations, these enzymes likely replaced an ancestral bifunctional fructose 1,6-bisphosphate aldolase/phosphatase enzyme involved in gluconeogenis [78].

Finally, early enzymes probably had a more relaxed substrate specificity than in modern metabolism. This reasoning is the basis of “The Game of the Pentose Phosphate Cycle” study where Meleindez-Hevia and Isidoro showed that generic aldolases and ketolases could generate a large set of sugar phosphate interconversions and the subsequent growing specificity of the enzymes lead to a minimal solution, that in fact is equivalent to the naturally occurring pathway in *E. coli* to recycle pentoses to hexoses [79]. Similarly, Noor *et al* expanded contemporary central carbon metabolism by assuming a relaxed specificity of enzymes [80]. With this methodology, they showed that the central carbon metabolism in *E. coli* connects input sugars and the key precursors metabolites essential for biomass and energy production by the minimal number of enzymes, suggesting that contemporary metabolism is a small subset of the original possibilities.

The metabolic network of *E. coli* represents the result of billions of years of catalytic refinement through natural variation and natural selection. However some of the properties germane to early evolution are common to all life forms and should still be at least partially conserved and, it is generally of interest to know whether the best-studied metabolic system is an RAF. With a few restrictions, for example the introduction of generic catalysts where no cofactors are involved, it indeed is. This is an encouraging result for future studies on the metabolic networks of ostensibly more primitive organisms such as acetogens and methanogens [44,81] whose carbon and energy metabolism is not only simpler than that of *E. coli*, but also more similar to chemistry at hydrothermal vents, and whose metabolism furthermore involves a more prominent role of catalysis by metals [46,82].

The critical role of cofactors in the *E. coli* RAFs might point to an interesting aspect of early chemical evolution. We see here that the size, hence in some respects the complexity, of RAFs within the *E. coli* metabolic network are dependent upon cofactors: a small number of catalysts that promote a large number of reactions each. Regardless of where life arose, in the very earliest phases of chemical evolution, there must have been both thermodynamically controlled reactions (the most stable compounds accumulate) and kinetically controlled reactions (the most rapidly synthesized products accumulate). By lowering the activation energy of a reaction, cofactors influence the latter class of reactions more than the former as seen in the PLP example before mentioned [20]. Hence the spontaneous, and perhaps inorganically catalyzed, synthesis of small amounts of a small number of organic cofactors at the onset of chemical evolution could have strongly influenced the nature of compounds that subsequently accumulated. With the advent of proteins, this principle might not have been discarded, depending on whether one interprets the *E. coli* metabolic map as harboring some relics from early evolution, or not. In our opinion, some of these original imprints may still be present in the metabolism of modern organisms, as seen by their recurrent use of the same set of metals and organic cofactors as catalysts of biologic reactions, a set much smaller than the number of protein families that have evolved to catalyze them. The directives of the continuity principle in evolution demand that complex biochemistries had to be preceded by simpler chemistries. Thus, the initial RAF set would certainly be much simpler than the one analyzed in this paper but migth have already manifest the principle of cofactor and metal dependencies as recurrently is observed across studies showing their central role in modern metabolism [11,59,83].

We have shown that the *E. coli* reaction network can produce useful insights into primordial RAFs by identifying the essential role of metals and molecules such as ATP, CoA or thiamin in metabolism. This suggests the existence of some sort of abiotic autocatalysis at the onset of primordial metabolic networks. We have also shown here that maximal RAFs can be efficiently detected in real biological data, but the identification of large irreducible RAFs and the minimal food sets required to support a maximal RAF remain challenging problems.

## Appendix

### Proof that min-F RAF and min-F Generation are NP-complete

First note that both problems are in the complexity class NP, since we can determine in polynomial time (in the size of the input) whether a set of reactions is *F*^′^−generated and/or an RAF, for any given set *F*^′^.

Next, notice that it suffices to show that the simpler problem ‘min-F generation’ is NP-complete, since for any instance *I* of ‘min-F generation’ there is a corresponding instance *I*^′^ of ‘min-F RAF’ obtained by (i) making every molecule in *X* catalyze *every* reaction in , and (ii) taking ${\mathcal {R}}={\mathcal {R}}^{\prime }$ and *X* to be the molecules in the support of ${\mathcal {R}}^{\prime }$; under this correspondence, *I*^′^ has an affirmative answer if and only if *I* does.

We will show that ‘min-F generation’ is NP-complete by exhibiting a (polynomial-time) reduction from the following set-theoretic decision problem.

**Exact cover by 3-sets (X3C)**

INSTANCE: Finite set *Y* with |*Y*|=3*q*, *q* an integer; collection *S* of 3-element subset of *X*.

QUESTION: Does *S* contain an exact cover for *Y*, i.e., a subcollection *S*^′^ of *S* such that every element of *Y* occurs in exactly one member of *S*^′^?

The decision problem X3C was one of the early ones to be shown NP-complete by Karp [84]. We may assume, without loss of generality, that in X3C every element of *Y* occurs in at least one element (3-element subset) of *S*. As an example of X3C, consider the set *Y*={*a*,*b*,*c*,*d*,*e*,*f*} and

$$S = \{\{a,b,c\}, \{a,d,e\}, \{d,e,f\}\}. $$

In this case *S*^′^={{*a*,*b*,*c*},{*d*,*e*,*f*}} is the (unique) subset of *S* that provides an exact 3-cover for *Y*.

Given an arbitrary instance (*Y*,*S*) of X3C, we will construct an associated set ${\mathcal {R}}_{(Y,S)}$ of reactions. The set of molecules *X* involved in these reactions is the (disjoint) union *Y*∪*S*∪*W*, where *W*:={*w*_*s*_:*s*∈*S*}. Thus |*X*|=|*Y*|+|*S*|+|*W*|=3*q*+2|*S*|. Moreover, we will take the food set *F* to consist of *all* of *X*.

We next describe the reactions. First impose an arbitrary total order < on *Y*. Then for each set *s*={*a*,*b*,*c*}∈*S*, with *a*<*b*<*c*, consider the set ${\mathcal {R}}_{s}$ consisting of the following three reactions:

$$s \rightarrow a+b+c $$

$$a+b \rightarrow w_{s} $$

$$w_{s} + c \rightarrow s $$

Then define ${\mathcal {R}}_{(Y, S)} := \bigcup _{s \in S} {\mathcal {R}}_{s}$, and observe that ${\mathcal {R}}_{(Y, S)}$ is *F*−generated, and that $|{\mathcal {R}}_{(Y, S)} | = 3|S|.$

In the example above, with *s*={*a*,*b*,*c*},*s*^′^={*a*,*d*,*e*} and *s*^″^={*d*,*e*,*f*}, and with the molecules ordered alphabetically, ${\mathcal {R}}_{(Y, S)}$ comprises the nine reactions:

$$s \rightarrow a+b+c, a+b \rightarrow w_{s}, w_{s} + c \rightarrow s; $$

$$s' \rightarrow a+d+e, a+ d \rightarrow w_{s^{\prime}}, w_{s^{\prime}} + e \rightarrow s'; $$

$$s^{\prime\prime} \rightarrow d+e+f, d+e \rightarrow w_{s^{\prime\prime}}, w_{s^{\prime\prime}} + f \rightarrow s^{\prime\prime}. $$

**Claim:** (*Y*,*S*) has an exact 3-cover, if and only if ${\mathcal {R}}_{(Y, S)}$ is *F*^′^−generated for some subset *F*^′^ of *F* of size at most *q*=|*Y*|.

#### *Proof of Claim*.

First, suppose that *S*^′^ is an exact 3-cover for *Y*. Then |*S*^′^|=*q* and every element of *Y* is generated by a reaction that has its (sole) reactant in *S*^′^. It follows that for every *s*∈*S* (say, *s*={*y*,*y*^′^,*y*^″^} with *y*<*y*^′^<*y*^″^) the associated reaction *y*+*y*^′^→*w*_*s*_ has reactants that can be generated from *S*^′^. This ensures, in turn, that the other associated reaction *w*_*s*_+*y*^″^→*s* can proceed. Consequently, all of *S* can be constructed, and so each of the reactions *s*→*a*+*b*+*c* for all *s*={*a*,*b*,*c*}∈*S* can also now proceed. In summary, the reactants of all reactions in ${\mathcal {R}}_{(Y, S)}$ can be generated by starting just with molecules in *S*^′^. Thus ${\mathcal {R}}_{(Y, S)}$ is *F*^′^−generated for any subset *F*^′^=*S*^′^ of *F*(=*X*) of size *q* that provides an exact 3-cover for *Y*.

Conversely, suppose that ${\mathcal {R}}_{(Y, S)}$ is *F*^′^−generated for some subset *F*^′^ of *F*(=*X*) of size at most *q*=|*Y*| (we will show that this implies that *S* contains an exact 3-cover). For each molecule *m* in *F*^′^ for which either:

- *m*=*y*∈*Y*, or
- *m*=*w*_*s*_∈*W*.

we proceed as follows. In case (i) select replace *y* by *s* for any *s*∈*S* that contains *y* (this is possible since we have assumed earlier, without loss of generality, that every element of *Y* is present in at least one element of *S*); in case (ii) we replace *m* by *s* (i.e. the same *s* appearing in *w*_*s*_). Then *s* generates *y* in case (i), and *s* generates the reactants required to generate *w*_*s*_ in case (ii). In this way we can replace *F*^′^ by a subset *S*^′^ of *S*, with

(1) $$  |S'| \leq |F'| \leq q  $$

and for which ${\mathcal {R}}_{(Y, S)}$ is *S*^′^−generated. Thus, it is possible to generate each element of *Y* by some series of reactions from ${\mathcal {R}}_{(Y, S)}$ starting with just the molecules in *S*^′^.

Now, the only reactions that generate molecules in *Y* are those that have a single reactant in *S*, and any *s*∈*S* that is the product of any sequence of reactions from ${\mathcal {R}}_{(Y, S)}$ requires that all three elements of *s* are either present or produced earlier in that sequence of reactions. It follows by an inductive argument that each molecule in *Y* must be able to be generated by just a single reaction from *S*^′^, and so *S*^′^ is a 3-cover for *Y*. This requires that 3|*S*^′^|≥|*Y*|, and so |*S*^′^|≥*q*,which, combined with Inequality (1), gives |*S*^′^|=*q*, and so (since |*Y*|=3*q*) *S*^′^ is an exact 3-cover for *Y*, as required.

## Additional file

Additional file 1
**Table S1.** List of the 123 molecules of the food set and their impact on the RAF size.
