# Supplementary material for: Autocatalytic sets in E. coli metabolism
Source: J Syst Chem. 2015 Apr 1;6(1):4. doi: 10.1186/s13322-015-0009-7 (PMC4429071; doi:10.1186/s13322-015-0009-7)
Supplement: Additional file 1 — Table S1. List of the 123 molecules of the food set and their impact on the RAF size. [file 13322_2015_9_MOESM1_ESM.docx]

| Additional file 1: Table S1. List of the 123 molecules of the food set and their impact on the RAF size. | | |
| --- | --- | --- |
| Molecule | Decrease in RAF size | Name |
| 14glucan[c] | 18 | 1,4-alpha-D-glucan |
| 23dappa[c] | 1 | 2,3-diaminopropionate |
| 2agpe120[c] | 3 | 2-acyl-sn-glycero-3-phosphoethanolamine (n-C12:0) |
| 2agpe140[c] | 3 | 2-acyl-sn-glycero-3-phosphoethanolamine (n-C14:0) |
| 2agpe141[c] | 3 | 2-acyl-sn-glycero-3-phosphoethanolamine (n-C14:1) |
| 2agpe160[c] | 3 | 2-acyl-sn-glycero-3-phosphoethanolamine (n-C16:0) |
| 2agpe161[c] | 3 | 2-acyl-sn-glycero-3-phosphoethanolamine (n-C16:1) |
| 2agpe180[c] | 3 | 2-acyl-sn-glycero-3-phosphoethanolamine (n-C18:0) |
| 2agpe181[c] | 3 | 2-acyl-sn-glycero-3-phosphoethanolamine (n-C18:1) |
| 2agpg120[c] | 3 | 2-acyl-sn-glycero-3-phosphoglycerol (n-C12:0) |
| 2agpg140[c] | 3 | 2-acyl-sn-glycero-3-phosphoglycerol (n-C14:0) |
| 2agpg141[c] | 3 | 2-acyl-sn-glycero-3-phosphoglycerol (n-C14:1) |
| 2agpg160[c] | 3 | 2-acyl-sn-glycero-3-phosphoglycerol (n-C16:0) |
| 2agpg161[c] | 3 | 2-acyl-sn-glycero-3-phosphoglycerol (n-C16:1) |
| 2agpg180[c] | 3 | 2-acyl-sn-glycero-3-phosphoglycerol (n-C18:0) |
| 2agpg181[c] | 3 | 2-acyl-sn-glycero-3-phosphoglycerol (n-C18:1) |
| 2ddecg3p[c] | 1 | 2-dodecanoyl-sn-glycerol 3-phosphate |
| 2hdec9eg3p[c] | 1 | 2-hexadec-9-enoyl-sn-glycerol 3-phosphate |
| 2hdecg3p[c] | 1 | 2-hexadecanoyl-sn-glycerol 3-phosphate |
| 2odec11eg3p[c] | 1 | 2-octadec-11-enoyl-sn-glycerol 3-phosphate |
| 2odecg3p[c] | 1 | 2-octadecanoyl-sn-glycerol 3-phosphate |
| 2tdec7eg3p[c] | 1 | 2-tetradec-7-enoyl-sn-glycerol 3-phosphate |
| 2tdecg3p[c] | 1 | 2-tetradecanoyl-sn-glycerol 3-phosphate |
| 3hcinnm[c] | 3 | 3-hydroxycinnamic acid |
| 3hpppn[c] | 1 | 3-(3-hydroxy-phenyl)propionate |
| acac[c] | 1 | Acetoacetate |
| acnam[c] | 2 | N-Acetylneuraminate |
| all-D[c] | 1 | D-Allose |
| anhgm4p[c] | 1405 | N-acetyl-D-glucosamine(anhydrous)N-acetylmuramyl-tetrapeptide |
| arab-L[c] | 2 | L-arabinose |
| arbt6p[c] | 2 | Arbutin 6-phosphate |
| arbtn-fe3[c] | 3 | Aerobactin |
| ascb6p[c] | 2 | L-ascorbate-6-phosphate |
| aso4[c] | 1 | Arsenate |
| butso3[c] | 3 | Butanesulfonate |
| ca2[c] | 31 | Calcium |
| cbi[c] | 4 | Cobinamide |
| cd2[c] | 1 | Cadmium |
| cgly[c] | 657 | Cys-Gly |
| chtbs6p[c] | 1 | Diacetylchitobiose-6-phosphate |
| cl[c] | 2 | Chloride |
| cobalt2[c] | 33 | Cobalt |
| cpgn[c] | 3 | Coprogen |
| crn-D[c] | 11 | D-carnitine |
| Molecule | decrease in RAF size | Name |
| cu2[c] | 18 | Copper (Cu^2+^) |
| cu[c] | 1 | Copper (Cu^+^) |
| cyan[c] | 1 | Hydrogen cyanide |
| cynt[c] | 1 | Cyanate |
| cys-D[c] | 1 | D-Cysteine |
| dmso[c] | 2 | Dimethyl sulfoxide |
| dmso[p] | 2 | Dimethyl sulfoxide |
| dopa[p] | 1 | Dopamine |
| ethso3[c] | 1 | Ethanesulfonate |
| f1p[c] | 1 | D-fructose 1-phosphate |
| fe3dhbzs[c] | 1 | Ferric 2,3-dihydroxybenzoylserine |
| fe3hox[c] | 3 | Fe(III)hydroxamate |
| fecrm[c] | 3 | Ferrichrome |
| feenter[c] | 4 | Fe-enterobactin |
| feoxam[c] | 3 | Ferroxamine |
| for[p] | 4 | Formate |
| fuc-L[c] | 2 | L-fucose |
| g3pc[c] | 4 | sn-glycero-3-phosphocholine |
| g3pi[c] | 1 | sn-glycero-3-phospho-1-inositol |
| g3ps[c] | 1 | Glycerophosphoserine |
| galct-D[c] | 1 | D-galactarate |
| galctn-D[c] | 2 | D-galactonate |
| galctn-L[c] | 6 | L-galactonate |
| galt1p[c] | 2 | Galactitol 1-phosphate |
| gbbtn[c] | 5 | γ-butyrobetaine |
| glc-D[p] | 2 | D-glucose |
| glcr[c] | 2 | D-glucarate |
| glyc2p[c] | 1 | Glycerol 2-phosphate |
| hg2[c] | 1 | Mercury |
| isetac[c] | 1 | Isethionic acid |
| k[c] | 166 | Potassium |
| lcts[c] | 1 | Lactose |
| lipoate[c] | 8 | Lipoate |
| lyx-L[c] | 2 | L-lyxose |
| mal-D[c] | 1 | D-malate |
| man6pglyc[c] | 1 | 2(α-D-Mannosyl-6-phosphate)-D-glycerate |
| melib[c] | 1 | Melibiose |
| mg2[c] | 1476 | Magnesium |
| mmet[c] | 1 | S-Methyl-L-methionine |
| mn2[c] | 145 | Manganese |
| mobd[c] | 28 | Molybdate |
| mso3[c] | 6 | Methanesulfonate |
| na1[c] | 4 | Sodium |
| ni2[c] | 11 | Nickel |
| no3[p] | 4 | Nitrate |
| Molecule | decrease in RAF size | Name |
| no[c] | 7 | Nitric oxide |
| o2[p] | 3 | Dioxygen |
| pacald[c] | 12 | Phenylacetaldehyde |
| peamn[p] | 1 | Phenethylamine |
| ppal[c] | 1 | Propanal |
| pppn[c] | 2 | Phenylpropanoate |
| progly[c] | 2 | L-prolinylglycine |
| rmn[c] | 2 | L-phamnose |
| sel[c] | 6 | Selenate |
| suc6p[c] | 1 | Sucrose 6-phosphate |
| sulfac[c] | 1 | Sulfoacetate |
| tartr-D[c] | 1 | D-tartrate |
| tartr-L[c] | 1 | L-tartrate |
| taur[c] | 1 | Taurine |
| thm[c] | 2 | Thiamin |
| tmao[c] | 2 | Trimethylamine N-oxide (cytoplasm) |
| tmao[p] | 2 | Trimethylamine N-oxide (periplasm) |
| tsul[c] | 1 | Thiosulfate |
| tungs[c] | 11 | Tungstate |
| tym[p] | 1 | Tyramine |
| xyl-D[c] | 2 | D-xylose |
| zn2[c] | 1432 | Zinc |
| atp[c] | 1518 | ATP |
| cbl1[c] | 3 | Cob(I)alamin |
| frulys[c] | 2 | Fructoselysine |
| fruur[c] | 3 | D-Fructuronate |
| glcn[c] | 5 | D-Gluconate |
| Glutathione | 30 | Glutathione |
| o2s[c] | 84 | Superoxide anion |
| Generic catalysts | | decrease in RAF size |
| genCat |  | 1367 |
| Protein |  | 1644 |
| RNA |  | 96 |
| spon |  | 42 |
| X |  | 1642 |
